# Supplementary figures and images for: A multi-objective based clustering for inferring BCR clonal lineages from high-throughput B cell repertoire data
Source: PLoS Comput Biol. 2022 Aug 29;18(8):e1010411. doi: 10.1371/journal.pcbi.1010411 (PMC9462827; doi:10.1371/journal.pcbi.1010411)

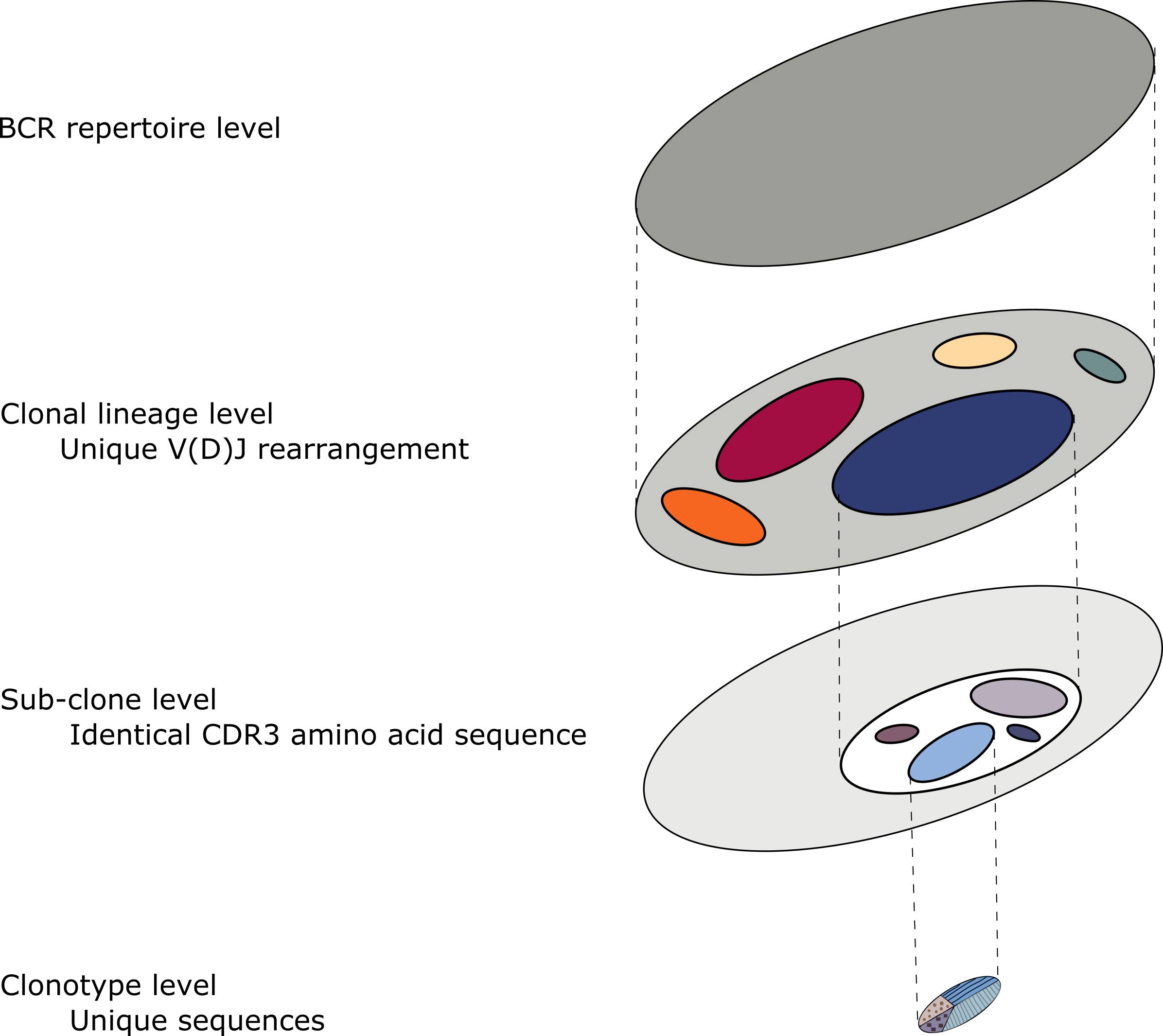

Supplement: S1 Fig — The first level represents the entire set of sequences without any grouping. The second level represents B cell lineages. Sequences within a clonal lineage have the same V(D)J rearrangement and evolved from a common ancestor. The third level groups clonally-related sequences with identical CDR3 amino acid content, forming a so-called sub-clone. The fourth level groups identical nucleotide sequences within a given sub-clone, termed as clonotype level. (TIFF) [file pcbi.1010411.s017.tiff]

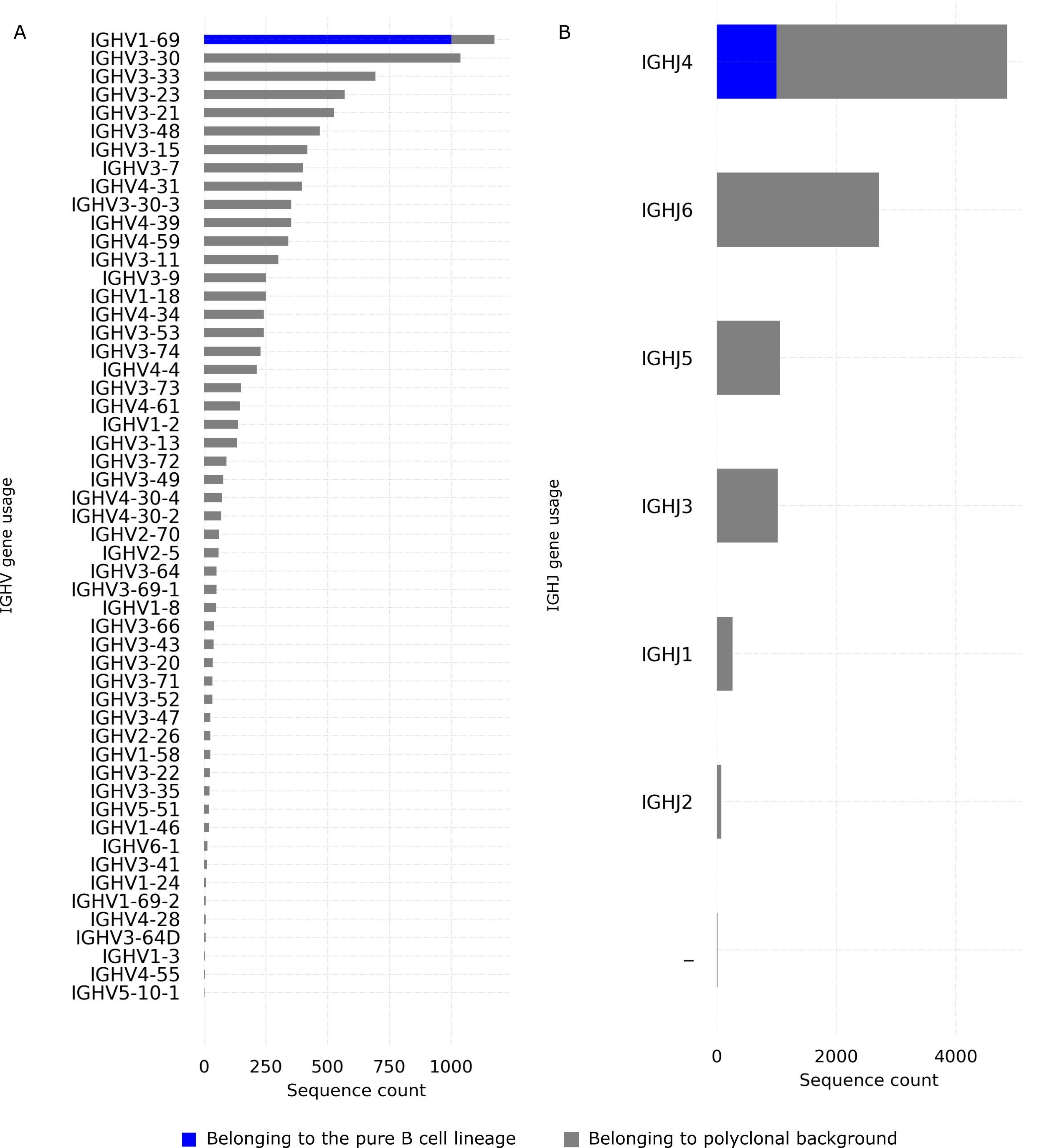

Supplement: S2 Fig — (A) The IGHV gene usage. (B) The IGHJ gene usage. The polyclonal background is in gray and the gene segment of AMR1 is shown in blue. (TIFF) [file pcbi.1010411.s018.tiff]

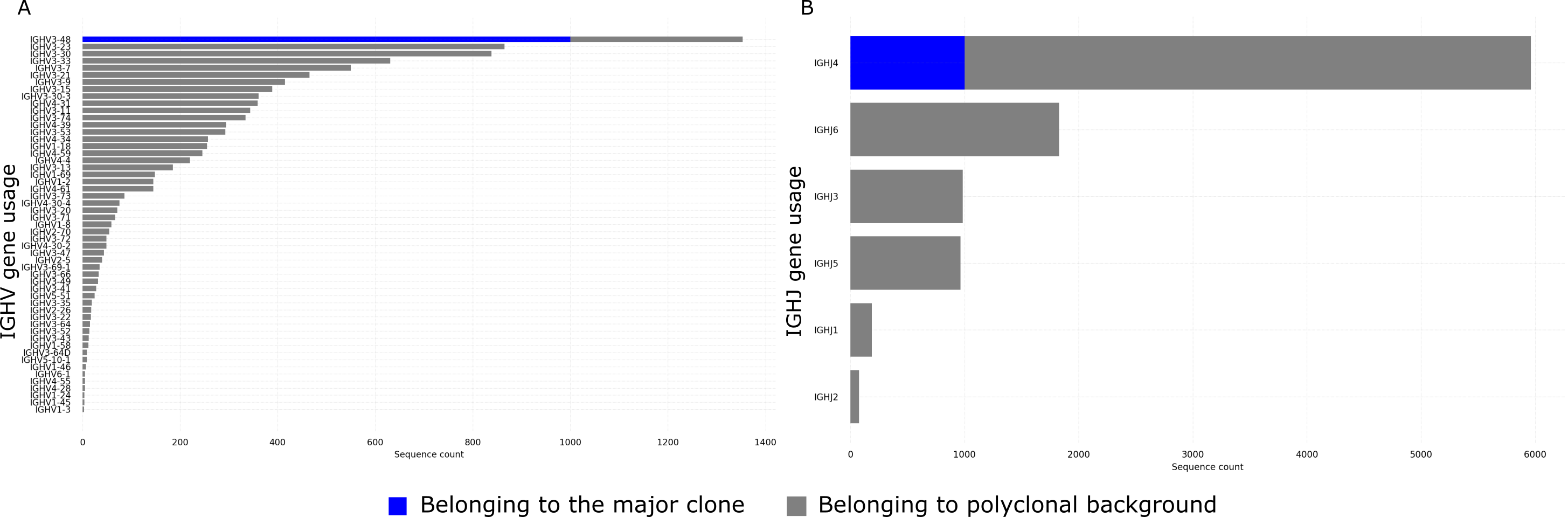

Supplement: S3 Fig — (A) The IGHV gene usage. (B) The IGHJ gene usage. The polyclonal background is in gray and the gene segment of AMR2 is shown in blue. (TIFF) [file pcbi.1010411.s019.tiff]

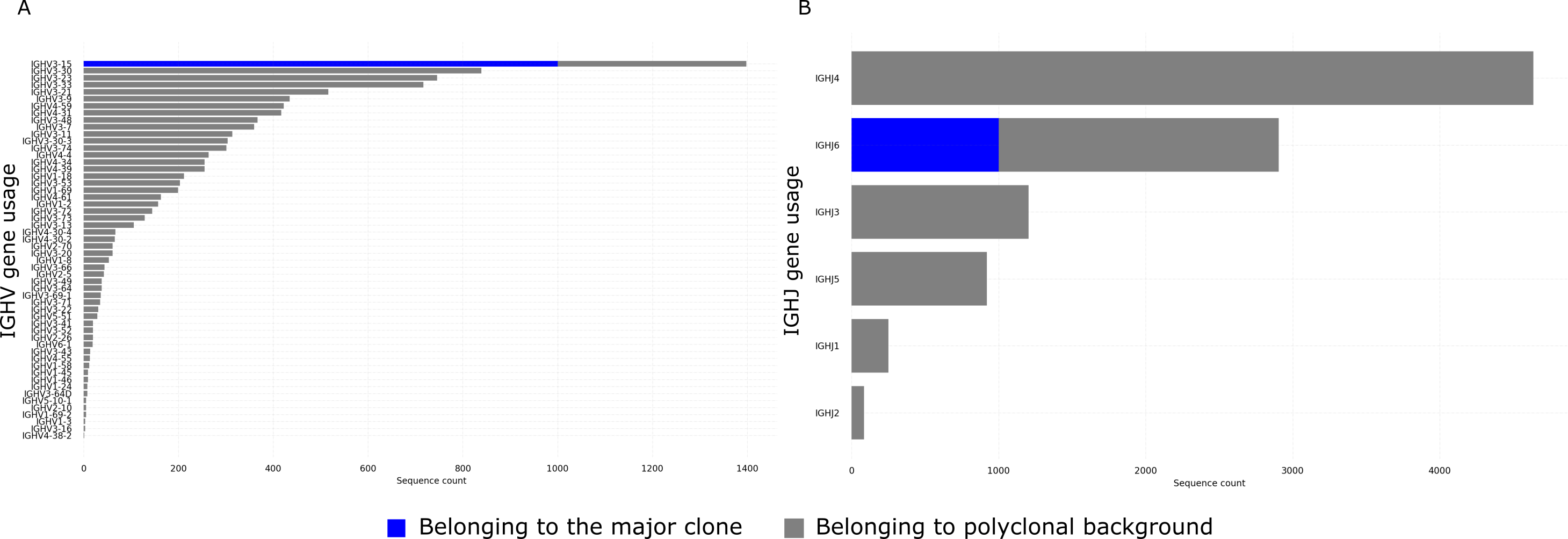

Supplement: S4 Fig — (A) The IGHV gene usage. (B) The IGHJ gene usage. The polyclonal background is in gray and the gene segment of AMR3 is shown in blue. (TIFF) [file pcbi.1010411.s020.tiff]

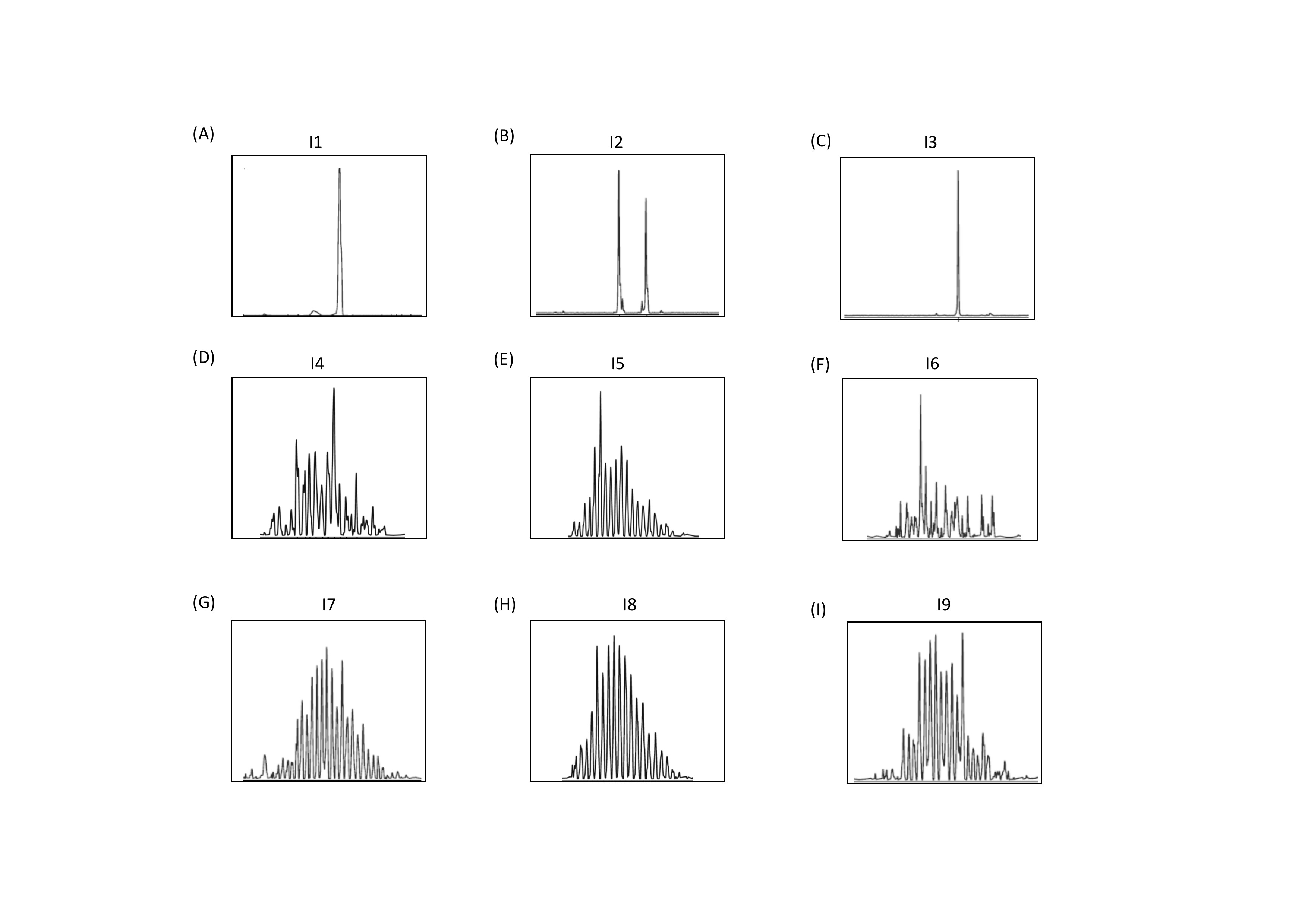

Supplement: S5 Fig — IGH-VDJ rearrangements were amplified using conventional methods and PCR products were further analyzed by capillary electrophoresis. (A-C) Samples from individuals with monoclonal B cell malignancy: monoallelic profile (A and C) or biallelic profile (B); (D-I) non-malignant samples: regular polyclonal profile (D, E, G, H, I) or irregular polyclonal profile (F). (TIFF) [file pcbi.1010411.s021.tiff]

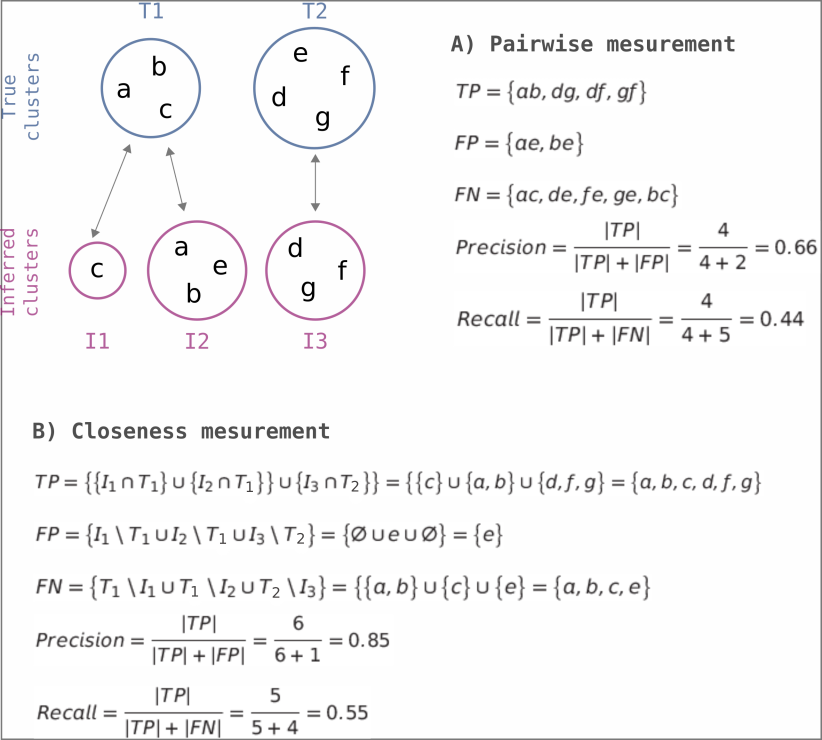

Supplement: S6 Fig — A) Pairwise B) Closeness. (TIFF) [file pcbi.1010411.s022.tiff]

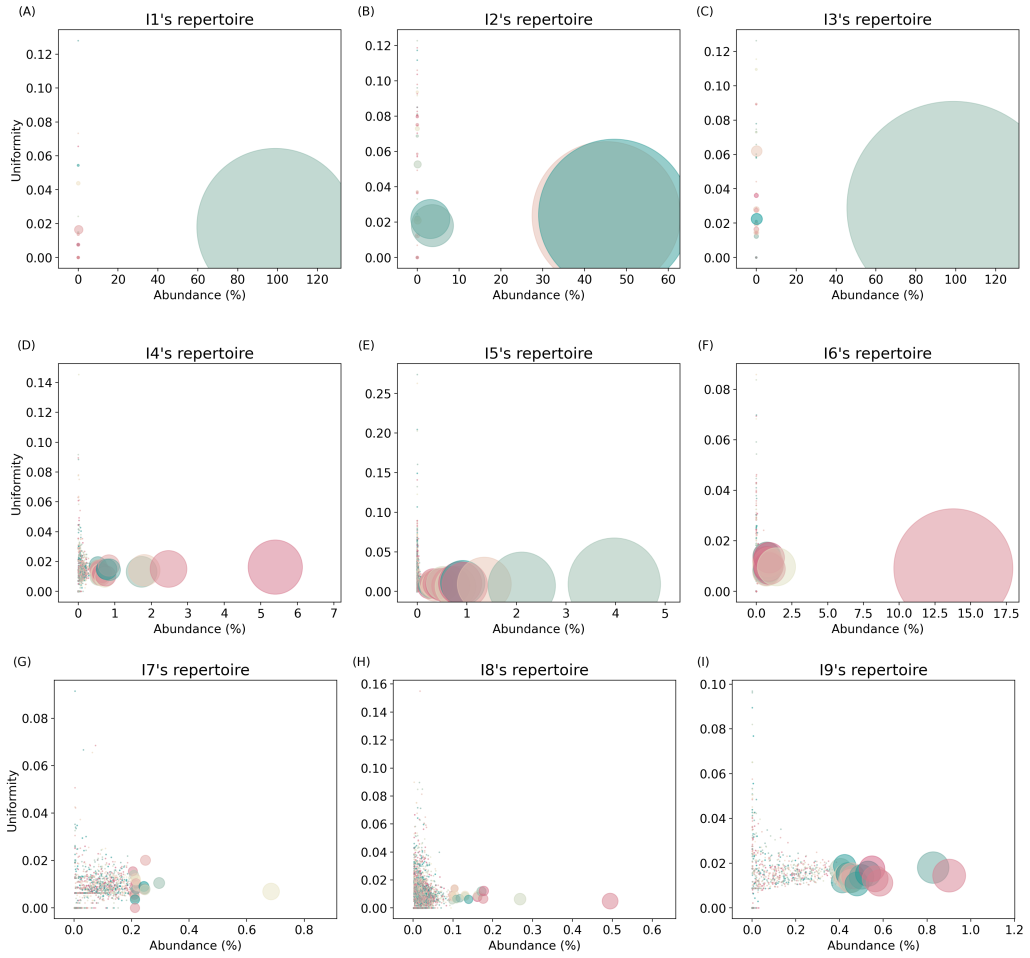

Supplement: S7 Fig — Each circle symbolizes a clonal lineage, and the circle area its abundance. The ordinate represents cluster uniformity Eq 2, while the abscissa the clonal lineage abundance in %. (A) I1, (B) I2, (C) I3, (D) I4, (E) I5, (F) I6, (G) I7, (H) I8, and (I) I9. (TIFF) [file pcbi.1010411.s023.tiff]

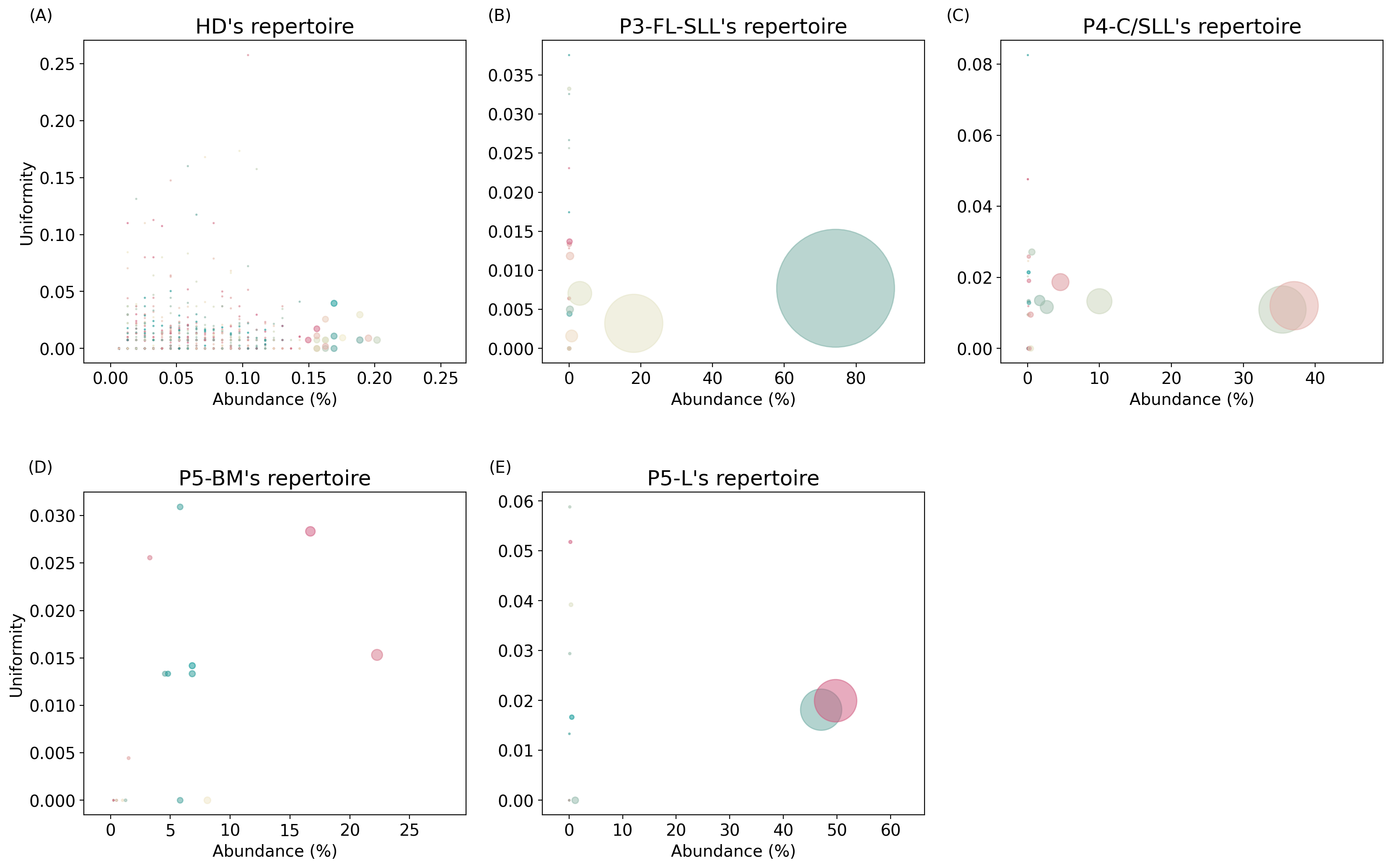

Supplement: S17 Fig — Each circle symbolizes a clonal lineage, and the circle area is proportional to clonal group abundance. The ordinate represents cluster uniformity (Eq 2), while the abscissa the clonal lineage abundance in %. (A) HD, (B) P3-FL-SLL, (C) P4-C/SLL, (D)P5-BM, and (E) P5-L. Report to Table 3 for repertoires’ properties and individuals’ labels. (TIFF) [file pcbi.1010411.s033.tiff]

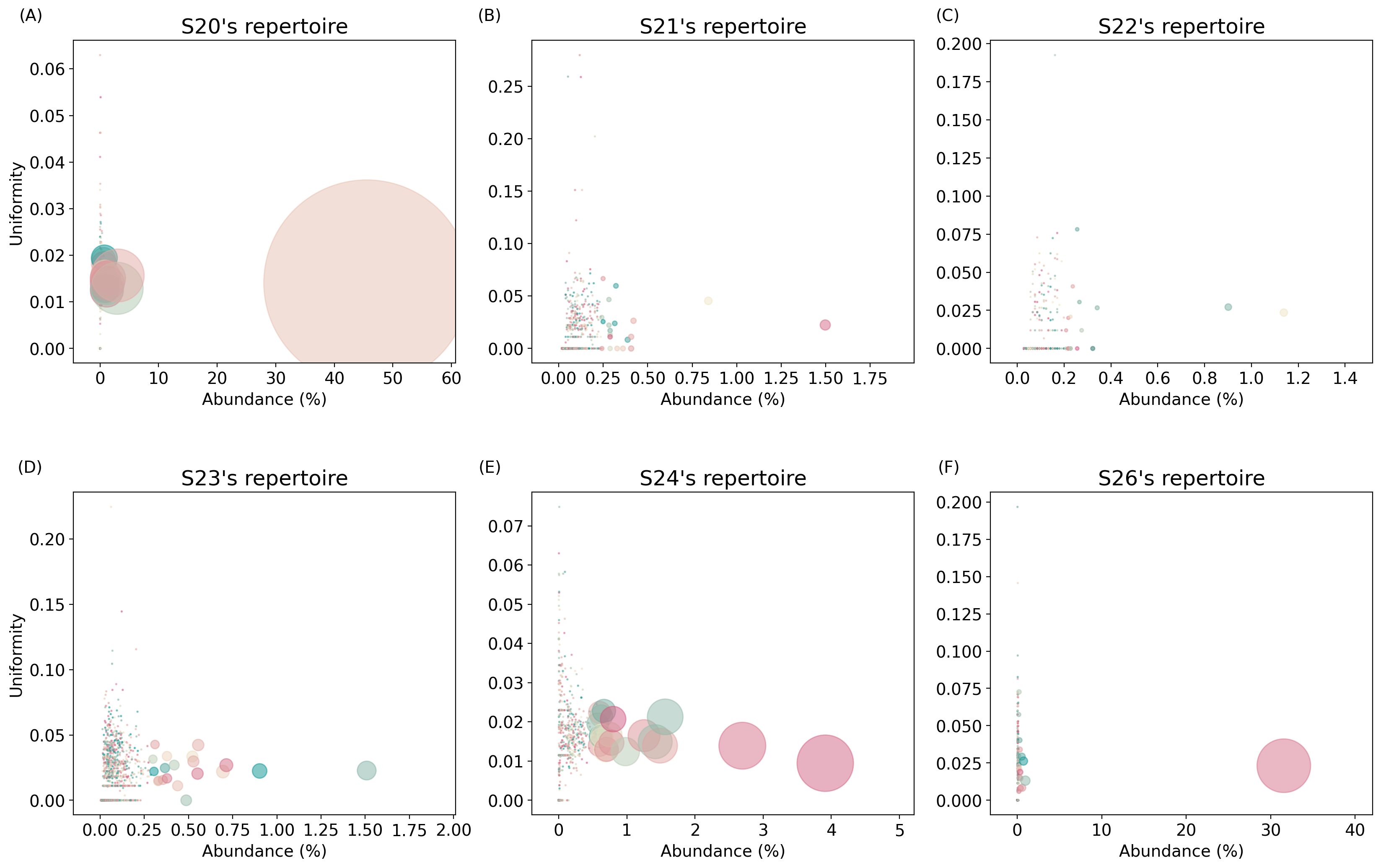

Supplement: S18 Fig — Each circle symbolizes a clonal lineage, and the circle area is proportional to clonal group abundance. The ordinate represents cluster uniformity (Eq 2), while the abscissa the clonal lineage abundance in %. (A) S20, (B) S21, (C) S22, (D) S23, (E) S24, and (F) S26. Report to Table 4 for repertoires’ properties and individuals’ labels. (TIFF) [file pcbi.1010411.s034.tiff]

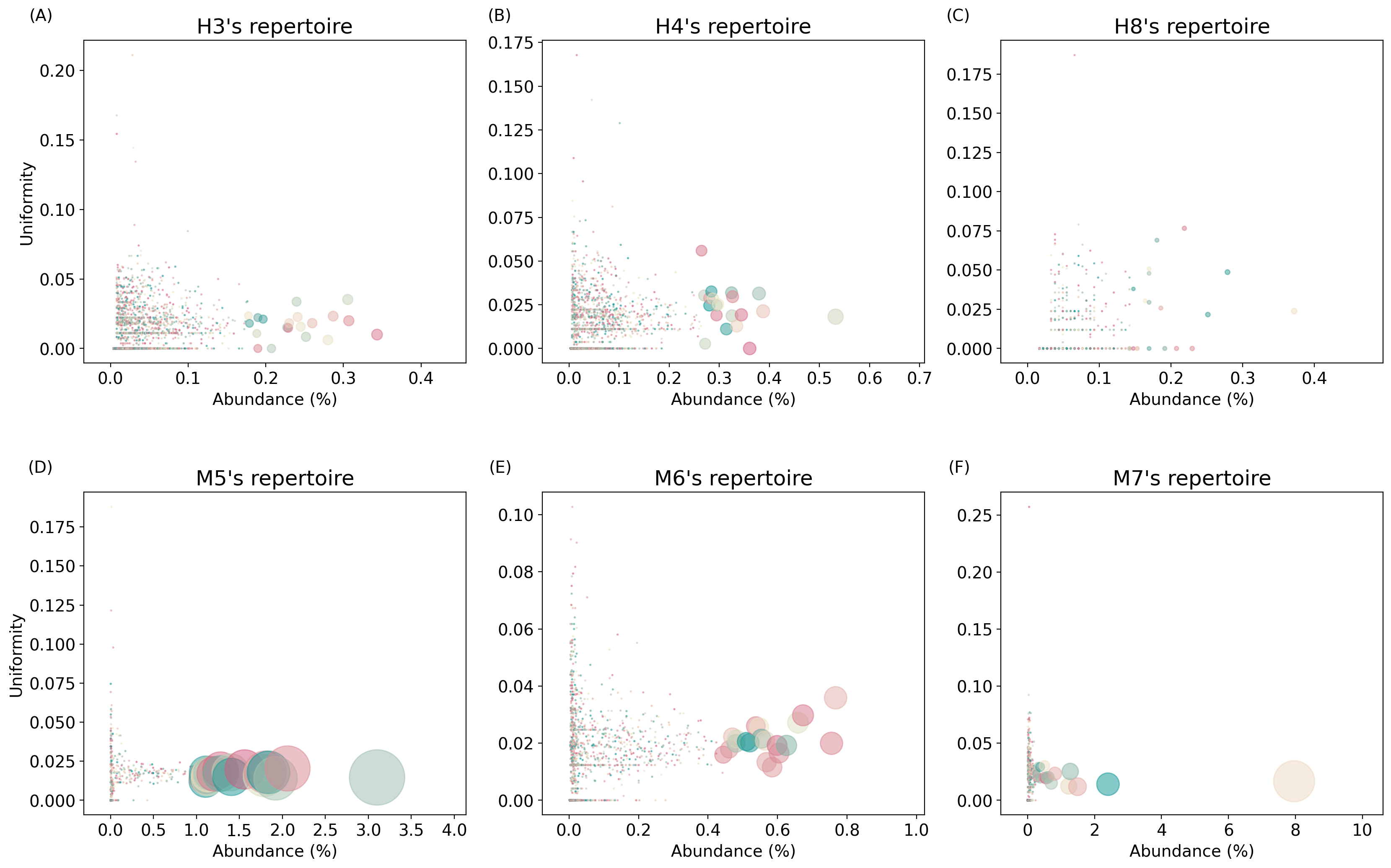

Supplement: S19 Fig — Each circle symbolizes a clonal lineage, and the circle area is proportional to clonal group abundance. The ordinate represents cluster uniformity (Eq 2), while the abscissa the clonal lineage abundance in %. (A) H3, (B) H4, (C) H8, (D) M5, (E) M6, and (F) M7. Report to Table 4 for repertoires’ properties and individuals’ labels. (TIFF) [file pcbi.1010411.s035.tiff]
