# Supplementary material for: A multi-objective based clustering for inferring BCR clonal lineages from high-throughput B cell repertoire data
Source: PLoS Comput Biol. 2022 Aug 29;18(8):e1010411. doi: 10.1371/journal.pcbi.1010411 (PMC9462827; doi:10.1371/journal.pcbi.1010411)
Supplement: Attachment — Submitted filename: Response-Reviewers.pdf [file pcbi.1010411.s036.pdf]

Comments of the PLoS computational biology

[1] **A letter containing a detailed list of your responses to the review comments and a description of the changes you have made in the manuscript.** Please note while forming your response, if your article is accepted, you may have the opportunity to make the peer review history publicly available. The record will include editor decision letters (with reviews) and your responses to reviewer comments. If eligible, we will contact you to opt in or out.

[2] **Two versions of the revised manuscript: one with either highlights or tracked changes denoting where the text has been changed; the other a clean version (uploaded as the manuscript file**).

Reviewer #1:

The overall goal of this manuscript is to provide an algorithm whereby, before BCR diversity and sub-categories are established, very closely related sequences are grouped. This approach would presumably add more power to the final goal of mapping the gross clustering results or the distinctions onto a given target, for example, outcomes from a disease or infection. Overall, this goal is new and will be a very useful contribution to the field. (Algorithm 1 figure is particularly useful and clear.)

Here are some minor issues.

1. Authors seem to go back and forth between **clonal grouping** and **identical CDR3 junctions** versus **similarity of CDR3 junctions with the same V's and J's.** This seems to be particularly a problem in the **Introduction.** This reviewer believes **the term clonal usually represents identity to most readers**…Authors should consider reviewing their language, especially in the introduction, and make **a clear distinction between identical sequences, from V to CDR3 to J versus closely related sequences that may indeed be targeting the same epitope.**

   r: we agree that the introduction was not clear and sometimes ambiguous. To clarify, we rewrote the introduction and added a Figure in the supplementary file (Fig. S1) to explain better the terms used in our work. We defined the main goal of our work and justified why we focus on BCR clonal lineage grouping.

2. With regard to number 1 above, are the authors trying to indicate that a **V-CDR3-J defines the clones** and **the somatic hyper-mutation divergence** is what is the basis for **"intra-clonal grouping"**? Once again, if so, that may need to be written out more carefully. Except, this preceding consideration may not be accurate because authors refer to **clonal grouping based on CDR3 lengths rather than exact amino acid sequences**?

r: we hope that the new introduction and the Figure S1 can clarify this point. We believe that BCR repertoires can be grouped on different levels, as illustrated in Figure S1. The first level represents the entire set of sequences without any grouping. The second level represents B cell lineages, where sequences of a given clonal lineage have the same V(D)J rearrangement and evolved from a common ancestor. The third level groups clonally-related sequences with identical CDR3 amino acid content, forming a so-called sub-clone. The fourth level groups identical nucleotide sequences within a given sub-clone, termed as clonoltype level.

3. Exactly which **kind of leukemia** does this phrase refer to: “Three of these samples contained clonal leukemic cells”? Do these **three leukemias represent a diagnosis of B-cell, acute lymphocytic leukemia**?

   r: These were chronic lymphocytic leukemia samples ; we changed the text to clarify this point.

4. The **Fig. 3, 4 legends should be divided into A-F (or A-I) with explanations for each panel.5.**

   r: we replaced Fig. 3 by a new Figure 4, and we added an entire section to discuss parameter optimization, see Section 3.1. Fig. 4 was replaced by a simplified one, see Figure 11.

5. It really seems like **at least one more experimental set should be evaluated**. If not, the **authors should discuss specific limitations**. For example, have the authors considered **the ireceptor.org repository** or a **collection from Adaptive Biotechnologies**?

   r: We thank the reviewer for providing this helpful reference. From the IReceptor repository, we selected 17 repertoires from two different published studies [35, 34]. The aim was to check whether MobiLLe could reproduce their results. From the first study, we selected five experimental repertoires of patients with different lymphoproliferative disorders, while from the second one, we selected 12 repertoires of healthy donors and patients with moderate/severe COVID-19. We present and discuss the results in Sections 3.3.2 and 3.3.3. MobiLLe achieved the same conclusions as both studies helping to identify clonal expansions in patients with different lymphoproliferative disorders and to detect immunological perturbations in severe/moderate Covid-19 disease.

Reviewer#2:

Abdollahi et al. developed a tool for BCR clustering based on a multi-objective clustering approach. The performance shown seems promising based on simulations and comparisons with similar methods. The strength of the manuscript is that simulations are well planned, with very detailed results reported in the results section.

The weakness of the manuscript is **the method itself** which seems **trivial**. Also, the **implementation** (the software provided in GitHub) has **limited options for parameter tuning or customization.**

r: we agree that MobiLLe had few parameters; thus, we added a set of new parameters to (i) choose among four different distances for IGHV, IGHJ, and CDR3, (ii) filter out sequences with low abundance or low quality, (iii) define coefficients to compute a ponderate mean instead just an arithmetic mean, (iv) run or not refinement and the 'merge singleton' steps. Parameter optimization is described in detail in Section 3.1, while parameter options are explained at GitHub. We hope to provide the community with a versatile method, easily adaptable to different research purposes.

Overall, I think the manuscript can be improved by potentially addressing the following questions:

(1) There is **no discussion** regarding why this paper should only **focus on BCR** and why the same method (multi-objective) clustering cannot be applied to **TCR clustering**.

r: As TCR sequences do not undergo somatic hypermutation (SHM); it is easier to identify clonally-related TCRs once identical sequences form a clonal lineage. On the other hand, upon antigen activation, B-cells undergo rapid proliferation and further diversification of their BCR sequences by SHM, introducing nucleotide substitutions into the BCR V regions. B cells for which SHM produced BCR with higher affinity for their cognate antigen expand, while those with a lower affinity are eliminated, thereby contributing to the affinity maturation of the B lymphocyte. This results in antigen-specific B-cell lineages with increased BCR affinity. Therefore a (theoretical) B-cell lineage includes the unmutated ancestor and all mutated variants making reconstruction more challenging than T-cell lineages. Thus, we focus on the BCR clonal lineage grouping task, which has scientific and clinical importance. We rewrote the introduction to clarify this point, giving our motivations for focus on BCR lineage clonal grouping.

(2) Although many methods are compared, there is a **lack of discussion or comparison** with another popular method for **multi-objective clustering based on the deep learning framework** (such as auto encoder, e.g., Nature Communications 12: 1605 (2021))

r: DeepTCR is a deep neural network that was used for classifying or grouping epitope-specific T cell receptor sequences. It is not the same question that MobiLLe addresses: the BCR clonal lineage grouping. Cernatelly, the method could be adapted to BCR data, but it requires retraining networks and considers specific features of BCR repertoires such as SHM. We thank the reviewer for this reference, and we intend to test/adapt MobiLLe to the epitope grouping task in a future work. We discuss this possibility in the Discussion section.

(3) There are other distance metrics such as **the geometric Isometry-based (implemented in GIANA)** has shown to achieve **much faster computational speed**. Is this one possible option for this implementation?

r: In this new version, we considered the GIANA distance metric, and also added k-mer based distance. Now, users can choose among four different ways to compute distance.

(4) It would really strengthen the paper if authors present their results on **real TCR reads** (such as the BCR profile called from TCGA, published by Liu Group) or **provide their tool as an online** resource where users **can upload their reads** for fast clustering analysis.

r: We added 18 new experimental repertoires, downloaded from the IReceptor repository, as suggested by the first reviewer (Section 3.3.2 and 3.3.3). We hope these additional analyses show the potential of MobiLLe in reproducing the results of previous works [34, 35], and analyze repertoires from different clinical conditions.

To answer the second point, we are actually working on an online resource to simplify MobiLLe usage. The web server is not yet published but the reviewer can access it from http://www.lcqb.upmc.fr/viclod - user : **viclod** - password : **cdr3SU2022!**

We ask to not diffuse the link since it is still a " work in progress" that will be submitted to publication.

Reviewer #3:

In this work, the authors present a clustering algorithm, MobiLLe, for B-cell immune repertoire datasets. It has multiple objectives and allows the refinement of clones by minimizing intraclonal distances and maximizing interclonal distances. They show MobiLLe's performance on synthetic data produced using GCTree and on experimental data. They also compare MobiLLe's performance to other clustering algorithms. Overall, the authors show a

promising, interesting algorithm, but I don't know how it will work on most datasets without being able to handle singleton clones.

Major comments:

Equation 1 presents the distance between sequences. The authors stated **the distance between V genes takes on a binary value whereas J genes do not**. There are quite a few V genes that are very similar to each other (at least 90% similar in normalized Hamming distance using nucleotides). Moreover, some V genes are quite synonymous with one another, possibly differing in, say, only one amino acid. I have **two questions**. What motivated the **choice for the binary measure for V gene distances**? How does **the performance change using a Levenshtein distance** measure as is or one **which weights changes in the framework and complementarity determining regions differently**?

r: we increased the MobiLLe parameter setting. Now, it is possible to choose the distance type for IGHV as well as IGHJ, and CDR3. We implemented four distinct types: binary, Leveinsten, k-mers and GIANA. We discuss the parameters configuration in Section 3.1.

While the authors detail the properties of the monoclonal repertoires, it's not clear to me what the **polyclonal repertoire is composed of, i.e., how different is the background from the signal**. Could the authors have a figure indicating, at the very least, **how the V gene, J gene, and CDR3 distributions of the background repertoires appear?** I'm imagining six plots. Three plots showing the statistics at the level of MobiLLe's clustering using lineage counts and three plots showing the statistics at the level of unique counts for each sequence (better yet, unique counts for each non singleton sequence if that abundance information is available). On these plots, could they please indicate the signal V and J genes and CDR3 length, so that everything is summarized in one figure.

r: As suggested by the reviewer, we computed distributions of the background repertoires and added them in the Supplemental Information Figures 2, 3 an 4.

How does MobiLLe compare to hierarchical clustering alone? While comparing these other algorithms is useful, I don't have a sense of how much refinement MobiLLe is performing compared to its initial step.

r: We thank the reviewer for raising this important issue. We did not compare MobiLLe to hierarchical clustering alone because Nouri and Kleinstein et al. [14] showed that Scope outperforms hierarchical clustering. Thus, we kept only the last version of the authors, that is Scope.

In order to show how much refinement step improve MobiLLe performance, we carried out an exhaustive parameter variations (Section 3.1). Figure 5.A shows the importance of using refinement steps, as it systematically improved the clustering accuracy.

Fixed hierarchical clustering thresholds are typically chosen at **85% or 90% based on the bimodal features apparent from the normalized Hamming distance of nearest neighbors in a V gene, J gene, and CDR3 length bin.** Because of this, I'm very curious how MobiLLe can be used with **higher CDR3 similarity in the pre-clustering step.** In practice, I would expect **many singleton clones because B-cell immune repertoires are highly undersampled**. Besides getting MobiLLe to work ??, how can the authors motivate **choosing lower similarity thresholds either statistically or biologically**? What are the **impacts in analyses of precluding singleton clones from existing?** Might the authors be able to implement this by the next iteration of reviews?

r: We implemented an extra algorithm in the refinement step for merging singletons when it improves cluster's uniformity, see Section 2.1.2 and algorithme 2. Then, we showed that merging singletons improves substantially clustering accuracy, see Section 3.1 and Figure 5.B.

Minor comments:

Fig 4: Because so many circles overlap, what's being presented is obscured. Is it possible to coarse-grain the information being presented? Unless I don't understand what's being presented, could the authors simply present log PDF vs. log clone abundance with a histogram, line, or scatter plot?

R: We replaced this Figure by a simplified one, see Figure 11. Our purpose is to give a rapide preview of the type of repertoire (monoclonal, oligoclonal, and polyclonal) and the number of expanded clonal lineages. Further details can be explored using the standard MobiLLe output plots, shown in the Supplemental Information..

Fig 5: I don't know what the "agreeable" label means. Is this MobiLLe's performance?

r: This was a typographical error. We changed the legend of that Figure by replacing the name "Agreeable" by "MobiLLe", see Figure 7.

Fig S1: Axis labels? I don't know what's being shown

r: The axis labels have been added

Fig. S2: There is no T3 in the diagram. In the main text and in the equations, T3 shows up.

r: done
