# Supplementary material for: A multi-objective based clustering for inferring BCR clonal lineages from high-throughput B cell repertoire data
Source: PLoS Comput Biol. 2022 Aug 29;18(8):e1010411. doi: 10.1371/journal.pcbi.1010411 (PMC9462827; doi:10.1371/journal.pcbi.1010411)
Supplement: Attachment — Submitted filename: ReviewerResponsesRevision2.pdf [file pcbi.1010411.s037.pdf]

Reviewer's Responses to Questions

**Comments to the Authors:**

**Please note here if the review is uploaded as an attachment.**

Reviewer #1: Authors successfully addressed all of my concerns.

Reviewer #2: All my questions are properly addressed.

Reviewer #3: Thank you to the authors for their revisions. The authors have addressed my concerns.

Minor comments:

Please read through your manuscript thoroughly. There are some typos that cause a lot of confusion. Thanks.

r: we corrected as many as possible grammar and orthography errors.

Algorithm 2: Is the b_i line necessary? b_i doesn't appear to be used in this algorithm.

r: we corrected the algorithm and we added some comments to clarify this point..

Fig. 2: Change S14 to S13 in panel B.

r: we modified the Figure accordingly.

Fig. 11: Should the y-axis read "Gini index" or "abundance"?

r: It is the abundance, we modified the Figure legend accordingly.

Fig 13: Panel C's y-axis should read "HCDR3 length [nt]" or something like that, no? Please make sure everything that is labeled "abundance" is actually abundance.

r: we modified the Figure accordingly.

Line 780: "Fig S19 and S19" to "Fig S18 and S19"

r: we modified the text accordingly.
